# Feasibility of Using PZT Actuators to Study the Dynamic Behavior of a Rotating Disk due to Rotor-Stator Interaction

**DOI:** 10.3390/s140711919

**Published:** 2014-07-07

**Authors:** Alexandre Presas, Eduard Egusquiza, Carme Valero, David Valentin, Ulrich Seidel

**Affiliations:** 1 Centre de Diagnòstic Industrial I Fluidodinàmica, Universitat Politècnica de Catalunya, Av. Diagonal, 647. ETSEIB. Pab. D+1. 08028 Barcelona, Spain; E-Mails: egusquiza@mf.upc.edu (E.E.); valero@mf.upc.edu (C.V.); david.valentin@mf.upc.edu (D.V.); 2 Voith Hydro Holding GmbH & Co. KG, Alexanderstrasse 11, 89522 Heidenheim, Germany; E-Mail: Ulrich.Seidel@voith.com

**Keywords:** rotating disk, PZT actuator, RSI, rotating excitation, resonance

## Abstract

In this paper, PZT actuators are used to study the dynamic behavior of a rotating disk structure due to rotor-stator interaction excitation. The disk is studied with two different surrounding fluids—air and water. The study has been performed analytically and validated experimentally. For the theoretical analysis, the natural frequencies and the associated mode shapes of the rotating disk in air and water are obtained with the Kirchhoff-Love thin plate theory coupled with the interaction with the surrounding fluid. A model for the Rotor Stator Interaction that occurs in many rotating disk-like parts of turbomachinery such as compressors, hydraulic runners or alternators is presented. The dynamic behavior of the rotating disk due to this excitation is deduced. For the experimental analysis a test rig has been developed. It consists of a stainless steel disk (r = 198 mm and h = 8 mm) connected to a variable speed motor. Excitation and response are measured from the rotating system. For the rotating excitation four piezoelectric patches have been used. Calibrating the piezoelectric patches in amplitude and phase, different rotating excitation patterns are applied on the rotating disk in air and in water. Results show the feasibility of using PZT to control the response of the disk due to a rotor-stator interaction.

## 1. Introduction

The transverse vibration of rotating disks has been a relevant research topic for decades because the disks have various applications in engineering. Rotating disks are a common element in rotating machines such as circular saws, wafer cutting machines, disk brakes, grinding wheels, data storage disks and turbine rotors. One of the cases of interest involving disk-like structures are the turbomachinery components with small gaps between rotating and static parts. In this case the perturbations originated by the static parts (guide vanes) superposed with the perturbations originated in the rotating parts (rotating blades) lead to a pressure pulsation known as rotor-stator interaction or RSI. RSI excites the rotating and the stationary part of the machine with a superposition of excitation patterns that depend on the number of harmonic, guide vanes and rotating blades and with a frequency content that depends also on the rotating speed of the rotor. RSI has been reported as the main cause of a critical failure when the rotating disk-like structure was rotating in air [1,2] and when it was rotating in water [3]. To avoid large vibration problems caused by the RSI, it is of paramount importance to determine the dynamic behavior of rotating disk-like parts, which involves the study of the natural frequencies and mode shapes of the structure and the study of the excitation characteristic.

Early work on the vibration of rotating disk was carried out by Campbell [4]. In this study the term “critical speed” at which the stationary wave appears on the disk was coined. The effect of rotation on the natural frequencies of the disk was introduced by Lamb and Southwell [5]. Their study was focused on a disk which rotated about its center with constant angular velocity. In [6] Southwell studied the vibration of circular disks clamped at their center. He considered the effect of a shaft that clamps the disk at its center, on the natural frequencies of the rotating disk. This effect was not considered in the previous study. Tobias and Arnold [7] used the linear plate theory to provide further explanation of the “stationary wave” effect. They concluded that the introduction of an imperfection reduced the magnitude of disk vibration under resonance conditions. Experimental studies with rotating disks have been carried out recently. Medigholi [8] showed the appearance of two resonances in the rotating disk (viewed from the rotating system) for each natural frequency of the disk stationary wave. In this case the disk was excited from the stationary frame. Ahn [9] could determine the mode shape of the rotating disk using two stationary sensors. He measured the phase difference between the signals of two proximity probes for this purpose. In this case, an electromagnet placed on the stationary system was the disk exciter. Some studies concerning rotating disks with different boundary conditions have been carried out by means of numerical simulation [10–13]. In [10] Heo studied the effect of the angular misalignment in the natural frequencies of the disk. Bauer [11] studied the effect of the attachment of the disk on the stationary part. Imperfections in bladed disks were considered by Pust [12,13]. Most of the mentioned cases deal with very thin disks that rotate in air or vacuum, but none of them studied the effect of rotation when the disk rotates with surrounding heavy fluids such as water. Kubota [14] studied this effect but only from the analytical point of view, so no experimental results were given.

The RSI phenomenon was described in [15,16], as a superposition of wake and potential effects. The combination of blades in the rotating part and guide vanes determine the excitation shapes that are applied in the machine [14]. Some recent studies have been developed studying this phenomenon [17,18], nevertheless all of them are centered in the study of the flow characteristics and not on the dynamic behavior of the structure itself. To study this effect from the rotating frame it is advantageous to use an excitation and measurement system placed on the rotating system (rotating disk-like part of the machine), since stationary sensors and actuators could affect the flow characteristics around the rotating part of the machine.

To excite the disk with a rotating excitation, light exciters (that do not affect the mass of the disk) have to be attached on the rotating frame. Because electromagnets or shakers are very heavy and may affect the mass of the structure, light and thin piezoelectric patches can be used in this case. PZTs are used in many cases as exciters [19–24]. Yang [19] studied the governing equation of an elastic plate due to the excitation of one PZT. He also modeled the dynamic behavior of the actuator. In [20], Gomis presented a control law for a piezoelectric actuator considering the hysteresis. Some studies have also been found with more than one acting piezoelectric patch. Cheng [21] placed several patches on a plate and studied the effect of the added mass of the actuators. In [22] Sekouri used piezoelectric patches to excite a thin circular plate. Finally, Wang [23] and Yan [24] studied the feasibility of suppressing the aerodynamic flutter of a rotating disk. In both studies the disk was rotating but the actuators were placed on the stationary frame. Although in some of the mentioned studies PZT actuators are used to excite a rotating disk, the exciters are placed in the stationary frame and no studies have been found with multiple PZTs actuators placed on the rotating structure acting as modal exciters. Furthermore, in the mentioned studies PZT actuators are used to excite very thin rotating disks (thicknesses of less than 1 mm), which is in the range of rotating disks such as CD drives, DVD drives and other data storage disks, and no studies have been found with PZTs actuators exciting thick and submerged disks in water.

In the present paper, the feasibility of studying the dynamic behavior of a rotating disk structure due to a rotor-stator interaction, by means of PZTs is analyzed. The main objective is to determine the dynamic response of a disk, rotating inside a casing with air or full of water, when it is excited with different excitation patterns that simulate the RSI. The remaining sections of this paper are organized as follows: Section 2 presents an analytical model that uses the thin plate theory for an annular plate, coupled with the pressure that the surrounding fluid exerts on the rotating disk to determine the natural frequencies and mode shapes and a one-dimensional modeling of the RSI. Section 3 describes the experimental setup used and the procedure to study this topic with PZT actuators. Section 4 includes numerical results for the amplitude of resonance of the first several natural frequencies of the rotating disk in air and in water and comparison between analytical and experimental method. Concluding remarks are given in Section 5.

## 2. Analytical Model7

Prior to the experimental tests with PZTs actuators exciting a rotating disk, an analytical model to predict the response of the rotating disk (in air and water) due to different rotating excitation patterns (that simulate the RSI) under resonance conditions, is presented.

### 2.1. Structural Response of a Rotating Disk in Air and in Water

**Figure 1.**
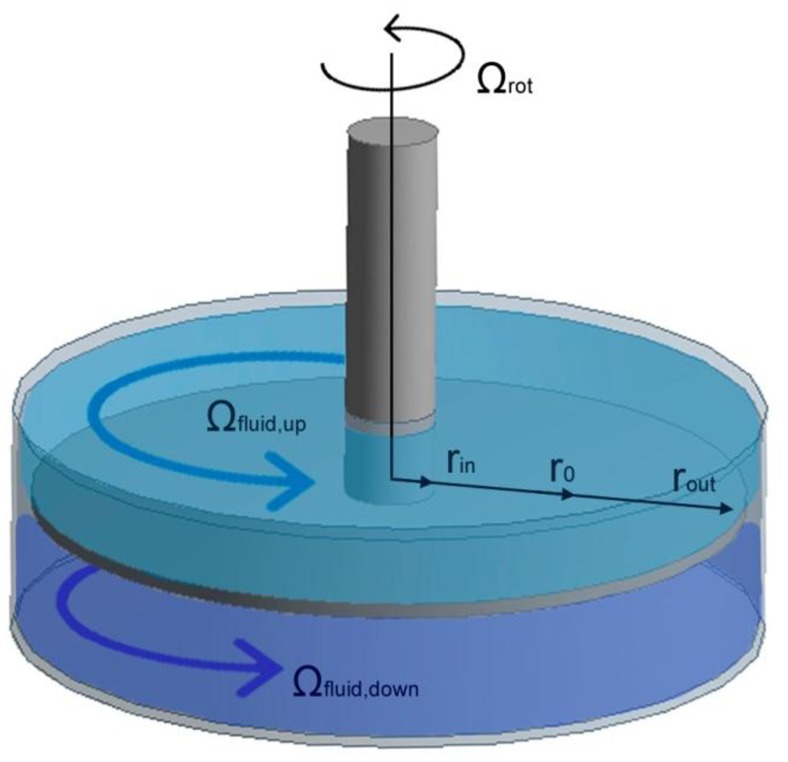
Model for a confined disk.

To study the natural frequencies and mode shapes of the rotating disk in air and in water, the model developed by Kubota [14] is used; particularized for a totally confined disk (Figure 1). The governing equation of the transverse vibration of a rotating disk in vacuum in cylindrical coordinates is:

(1) $$\rho_{D}h_{D}\frac{\partial^{2}w}{\partial t^{2}}+D{\left[\frac{\partial }{\partial r^{2}}+\frac{1}{r}\frac{\partial }{\partial r}+\frac{1}{r^{2}}\frac{\partial^{2}}{\partial \theta^{2}}\right]}^{2}w=0$$

*ρ_D_* and *h_D_* are the density and thickness of the disk respectively. *w* is the displacement of the disk in the axial direction. $\text{D}=\frac{Eh_{D}^{3}}{12\left(1-\upsilon^{2}\right)}$ is the bending stiffness of the disk, where E and υ the Young and Poisson modulus of the material.

For certain ratios *r_in_*/*r_out_* the first several natural frequencies correspond to mode shapes with no diametrical circles [25]. Considering only these mode shapes, which are also the more apt to be excited by the RSI [14], Equation (1) can be simplified for an annular disk, assuming that the vibration of the disk is uniform in the radial direction. In this case the vibration of the disk is studied in an averaged radius $r_{o}=\sqrt{r_{in}\cdot r_{\text{out}}}$. The surrounding fluid is considered taking into account the pressure that the fluid is applying on the disk surface at *r_o_* [14]:

(2) $$\rho_{D}h_{D}\frac{\partial^{2}w}{\partial t^{2}}+\frac{D^{*}}{{r_{o}}^{4}}\frac{\partial^{4}w}{\partial \theta^{4}}=p_{r_{o}}$$

In this Equation, D* is a parameter that depends on structural properties of the disk. Furthermore, for the considered mode shapes, the disk vibration at *r_o_* can be expressed as:

(3) $$w=\sum_{n=\pm 2}^{\infty }A_{n}e^{jn\theta }e^{j\omega_{n}t}$$

where |n| indicates the number of nodal diameters in the mode shape and the sign of n indicates the direction of the travelling wave that appears on the disk. ω_n_ is the corresponding natural frequency. In [14], Equations (2) and (3) are supposed to be valid also for the modes |*n*| = 1. Nevertheless, the discrepancy between experimental and analytical values for these modes shown in that study, suggest to include only the modes with |*n*| > 1, where the analytical model predicts with good accuracy the experimental values. To calculate *p_ro_*, it is assumed that the fluid on the tank is inviscid and incompressible. Therefore, a potential flow on the tank is considered. The solution of the potential function for each of the two fields in Figure 1 is given in [14]. Applying the Equation of Energy in the non stationary form [26], the term *p_ro_* in the disk frame can be expressed as:

(4) $$p_{r_{o}}=\rho_{F}r_{o}\sum_{n=\pm 1}^{\infty }\frac{A_{n}}{n}\cdot {\text{e}}^{\text{jn}\theta }\cdot {\text{e}}^{{\text{j}\omega }_{\text{n}}\text{t}}\left({\left(\omega_{n}+\text{n}\Omega_{\text{up}}\right)}^{2}\coth\left(\frac{{\text{nH}}_{\text{up}}}{{\text{r}}_{0}}\right)+{\left(\omega_{n}+n\Omega_{\text{down}}\right)}^{2}\coth\left(\frac{{\text{nH}}_{\text{down}}}{{\text{r}}_{0}}\right)\right)$$

*ρ_F_* is the density of the fluid and Ω_up_ & Ω_down_ is the rotating speed of the flow with respect to the disk (Figure 1). These two parameters depend on the viscosity of the fluid, on the geometry of the tank that contains the disk and on the speed of the disk. The dependence of Ω_rot_ with Ω_up_ and Ω_down_ is approximately linear [26]. Since Ω_fluid,up_ & Ω_fluid,down_ are very complex to obtain analytically (the calculus involves Navier-Stokes equations) a CFD calculation can be performed to obtain these two parameters.

When the surrounding fluid is air, since the density of the flow is very small, the term *p_ro_* can be neglected in comparison with the terms of Equation (2). Therefore, the natural frequencies for this case can be obtained as:

(5) $$\omega_{n,\text{air}}^{2}=\frac{n^{4}D^{*}}{\rho_{D}h_{D}r_{o}^{4}}$$

In [25] an alternative formulation to calculate the natural frequencies of an annular disk is proposed. Combining Equation (5) and the method proposed in [25] the value of D* can be obtained.

**Figure 2.**
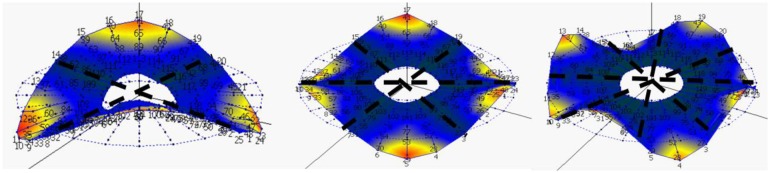
n = ±2, n = ±3, n = ±4 for the disk rotating in air, viewed from the rotating frame.

According to Equation (5), the natural frequencies of the rotating disk do not depend on the rotating speed, for slow velocities. In other studies [11], the dependence of the natural frequency on the rotating speed of the disk, viewed from the rotating system is studied. For slow velocities it is shown that only a very slight increase is observed. From Equation (5), it can be also seen, that a value of n-positive and n-negative will give the same positive solution of $\omega_{n,\text{air}}^{2}$. If the positive solution of n-positive and the positive solution of n-negative are introduced in Equation (3), it can be seen that the resulting mode shape, is a standing wave on the disk with n nodal diameters (Figure 2).

For the case that the surrounding fluid is water, Equations (3) and (4) are introduced in Equation (2), and the resulting Equation is obtained as:

(6) $$\begin{array}{l} \left(\left[\coth\left(\frac{nH_{\text{up}}}{r_{o}}\right)+\coth\left(\frac{nH_{\text{down}}}{r_{o}}\right)\right]\frac{\rho_{\text{water}}r_{o}}{n}+\rho_{D}h_{D}\right)\omega_{n,\text{water}}^{2}\\ +\left(\left[\coth\left(\frac{nH_{\text{up}}}{r_{o}}\right)2n\Omega_{\text{up}}+\coth\left(\frac{nH_{\text{down}}}{r_{o}}\right)2n\Omega_{\text{down}}\right]\frac{\rho_{\text{water}}r_{o}}{n}\right)\omega_{n,\text{water}}\\ +\left(-\frac{D^{*}}{{r_{0}}^{4}}n^{4}+[\coth\left(\frac{nH_{\text{up}}}{r_{o}}\right)n^{2}{\Omega_{\text{up}}}^{2}+\coth\left(\frac{nH_{\text{down}}}{r_{o}}\right)n^{2}{\Omega_{\text{down}}}^{2}]\frac{\rho_{\text{water}}r_{o}}{n}\right)\\ =0 \end{array}$$

From Equation (6), it can be seen that when the disk rotates, *Ω_up_* & *Ω_down_* are different than zero and this causes that the positive solution of *ω_n,water_* for n-positive is different than the positive solution of *ω_n,water_* for n-negative. This difference increases when *Ω_rot_* is increased (since *Ω_up_* & *Ω_down_* also increases [26]). Substituting the two solutions obtained for each n, in Equation (3), it is observed that two travelling waves travelling in opposite direction at different frequencies appear. The first one (n-positive and lower frequency) travels in the same direction as the rotating speed of the disk and the second one (n-negative and higher frequency) travels in the opposite direction [14].

A physical explanation for this effect is the influence of the added mass of the fluid on a forward wave and on a backward wave. According to [14], the free vibration of an annular disk is the superposition of a forward and a backward wave, for each diametrical mode n. For the annular disk with steady surrounding fluid, the added mass effect of this fluid on the forward and on the backward wave is the same and therefore both waves will have the same natural frequency and the corresponding mode shape at this frequency will be the superposition of both waves, which is a standing wave. With a relative rotation of the surrounding fluid with respect to the disk, the added mass effect will be different for the forward than for the backward wave, since the relative velocity of the fluid with respect to the wave will be different depending on the rotating direction of the wave. This causes, that the frequency of the backward wave will be different than the frequency of the forward wave. In this case, for each diametrical mode n a pair of natural frequencies, which correspond to the forward and to the backward wave, will appear on the disk. A similar effect is shown in [27], for a fluid-conveying pipe with periodic boundary conditions.

Increasing *Ω_disk_* will increase *Ω_up_* & *Ω_down_* and this will enhance the mentioned effect, which means to increase the difference between both natural frequencies. For higher values of *Ω_disk_* than considered in this paper, some terms may be included in Equation (1) [11] (due to centrifugal and Coriolis forces) and therefore the analytical solution would be modified. Furthermore, higher velocities of the disk leads to low pressure areas what could generate vapor bubbles (cavitation) [28,29], changing the added mass effect depending on the type and amount of cavitation [30]. Therefore, with the presence of cavitation, the solution of Equation (2) becomes much more complex.

The influence of the radial gap in the axial vibration is not considered in Equation (6). Nevertheless in [31] is shown that for short radial distances from the disk to the tank, the natural frequencies are decreased. In Equation (6), this effect can be considered increasing slightly *r_o_*, matching experimental and analytical results for the non rotating disk.

### 2.2. Rotor-Stator Interaction

**Figure 3.**
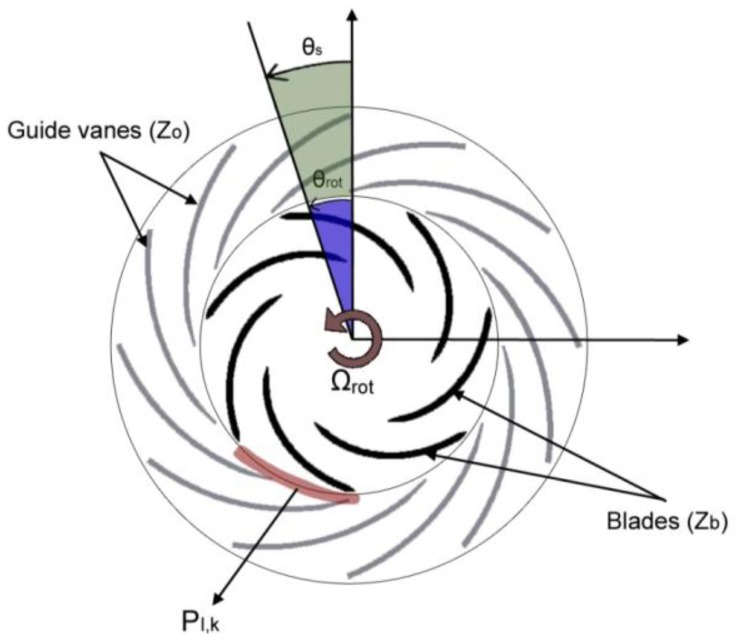
Rotor-stator interaction.

An unidimensional model for the Rotor-Stator Interaction is presented in [17]. When the rotating blades of the rotor pass in front of the static vanes of the stator (Figure 3) the pressure field in the gap between blades and vanes can be described as the superposition of all the combinations l,k:

(7) $$p_{lk}\left(\theta ,t\right)=A_{lk}\cdot \cos\left(lZ_{o}\theta_{s}+\emptyset_{l}\right)\cdot \cos\left(kZ_{b}\theta_{r}+\emptyset_{k}\right)\text{for}\;l=1,2,\ldots ,\infty$$

This pressure field can be viewed from the rotating frame or from the stationary frame. In this study, since the structural response is viewed from the rotating frame, the excitation will be also considered from the rotating frame. Transforming Equation (7) in the rotating coordinate (*θ_r_* = *θ_s_* − *Ωt*) this pressure field can be expressed as:

(8) $$\begin{array}{l} p_{l,k}=\frac{A_{lk}}{2}\cos\left(lZ_{o}\Omega_{\text{rot}}t-\left(kZ_{b}-lZ_{o}\right)\theta_{r}+\emptyset_{l}-\emptyset_{k}\right)\\ +\frac{A_{lk}}{2}\cos\left(lZ_{o}\Omega_{\text{rot}}t-\left(-lZ_{o}-kZ_{b}\right)\theta_{r}+\emptyset_{l}+\emptyset_{k}\right)\\ =\frac{A_{lk}}{2}\cos\left(lZ_{o}\Omega_{\text{rot}}t-\gamma_{1}\theta_{r}+\emptyset_{l}-\emptyset_{k}\right)+\frac{A_{lk}}{2}\cos\left(lZ_{o}\Omega_{\text{rot}}t-\gamma_{2}\theta_{r}+\emptyset_{l}+\emptyset_{k}\right) \end{array}$$

**Figure 4.**
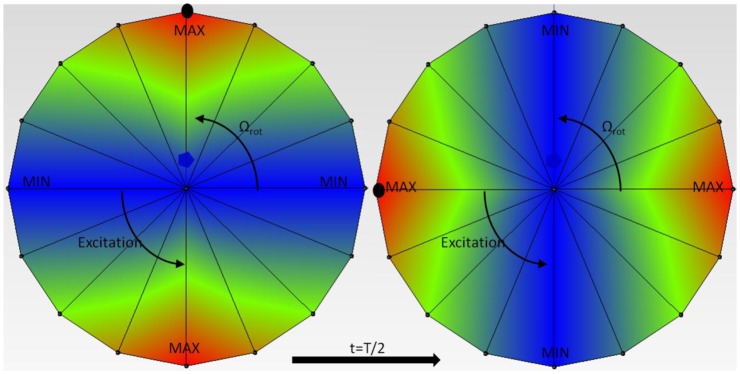
*γ* = +2 pressure pulsations for a rotating disk.

From Equation (8) it can be seen, that the excitation shape depends on the number of guide vanes and rotating blades (*γ*_1_ = *kZ_b_* − *lZ_o_*) and (*γ*_2_ = −*lZ_o_* − *kZ_b_*). This number represents the number of maximums and minimums in the pressure pulsation in a circle (Figure 4) and the sign of *γ*_1_ and *γ*_2_ indicates the rotating direction of the excitation. If it is positive, it indicates that the excitation rotates in the same direction (faster) than the rotating disk-like part. If it is negative it rotates in the opposite direction (slower).

Usually the term of the pressure pulsation that contains *γ*_2_ is not relevant for the RSI, since the several first mode shapes of a disk like structure have a small number of diametrical modes [17].

The excited frequency depends only on the number of guide vanes and on the rotating speed of the machine (*lZ_o_*Ω*_rot_*). For higher number of harmonics (l,k) lower amplitudes *A_lk_* are expected.

Rotating turbomachinery components that suffer the RSI, are designed to avoid resonances during their steady state. Nevertheless, during the acceleration or deceleration of the rotor, since the rotating speed changes, a resonance can occur if the natural frequency coincides with the excited frequency and the excitation shape with the mode shape.

### 2.3. Forced Response of the Disk Due to Rotor Stator Interaction

The frequency response function (FRF) is defined as the relationship between displacement at point ***p*** when a force is applied at point ***q*** for an arbitrary frequency ω. Considering the response in resonance of the mode n, *i.e.*, ω = ω_n_:

(9) $$H_{pq}\left(\omega_{n}\right)={\left[\begin{array}{ccc} h_{11} & \cdots & h_{1q}\\ \vdots & \ddots & \vdots \\ h_{p1} & \cdots & h_{pq} \end{array}\right]}_{n}=\frac{{\left\{X\right\}}_{n}}{{\left\{F\right\}}_{n}}$$

If only the response of the point 1 is studied:

(10) $$X_{1,n}={\left[\begin{array}{ccc} h_{11} & \cdots & h_{1q} \end{array}\right]}_{n}{\left\{\begin{array}{c} F_{1}\\ \vdots \\ F_{q} \end{array}\right\}}_{n}$$

#### 2.3.1. Air

When the disk is rotating in air at lower speeds, the mode shape is a standing wave on the disk (Equation (5) substituted in Equation (3)). It is considered, that the disk is excited in q equidistant points with respect to the origin of angles (point 1) and that the RSI pattern is equal in magnitude for all the q points (Equation (8)). The vector [*h*_11_ … *h*_1_*_q_*]*_n_*, is obtained for each of the considered natural frequencies ω_n_, using the information of the mode shape (in this case a standing wave). For the excitation {*F*}*_n_*, the pattern deduced in Equation (8) is introduced. Since the excitation changes its phase for each position (not all the points moving in phase or in counterphase), complex numbers have to be used to calculate the response [32]. For each of the considered natural frequencies:

(11) $$\begin{array}{cc} X_{1,n} & =\theta_{n}\cdot [\cos\left(n\left(\frac{0\cdot 2\pi }{q}\right)\right)\cdot \left(\cos\left(\gamma_{1}\left(\frac{0\cdot 2\pi }{q}\right)\right)-\text{j}\cdot \sin\left(\gamma_{1}\left(\frac{0\cdot 2\pi }{q}\right)\right)\right)\\ & +\cos\left(n\left(\frac{1\cdot 2\pi }{q}\right)\right)\cdot \left(\cos\left(\gamma_{1}\left(\frac{1\cdot 2\pi }{q}\right)\right)-\text{j}\cdot \sin\left(\gamma_{1}\left(\frac{1\cdot 2\pi }{q}\right)\right)\right)+\cdots \\ & +\cos\left(n\left(\frac{\left(q-1\right)\cdot 2\pi }{q}\right)\right)\\ & \cdot \left(\cos\left(\gamma_{1}\left(\frac{\left(q-1\right)\cdot 2\pi }{q}\right)\right)-\text{j}\cdot \sin\left(\gamma_{1}\left(\frac{\left(q-1\right)\cdot 2\pi }{q}\right)\right)\right)] \end{array}$$

Θ_n_ is an arbitrary complex constant that depends on the mode shape considered and j is the complex unity. From Equation (11), it can be deduced that to excite the structural mode ±n, the relationship between number of exciters q, excitation mode *γ*_1_ has to be the following:

(12) $$n=\pm \gamma_{1}\pm \lambda q$$

In Equation (12)*n*, *γ*_1_, *q* are entire and positive numbers that are defined by structural mode, excitation shape and number of equidistant exciters respectively and *λ* is an arbitrary entire number (including 0). From this equation it can be deduced, that the structural mode ±n is excited with one exciter whatever the excitation shape is, as for one exciter this is not defined. When when *γ*_1_ *=*±*n* the structural mode is excited with any number of exciters q. Otherwise, when *γ*_1_ *≠±n*, only for the number of exciters that accomplish Equation (12) the structural mode ±n is excited.

#### 2.3.2. Water

When the disk is rotating in water at lower speeds, two travelling wave appears for each n (Equation (6) substituted in Equation (3)). The same assumptions made for the excitation of the rotating disk in air are assumed now. The main difference in this case, is that the structural mode shape is a travelling wave and to express [*h*_11_ … *h*_1_*_q_*]*_n_* also complex numbers have to be used, since generally all the points are not moving in phase or in counterphase to each other. For each of the studied mode shapes, when *lZ_o_*Ω_rot_ = ω_n_:

(13) $$\begin{array}{l} X_{1,n}=\theta_{n}\cdot [\left(\cos\left(n\left(\frac{0\cdot 2\pi }{q}\right)\right)+j\cdot \sin\left(n\left(\frac{0\cdot 2\pi }{q}\right)\right)\right)\\ \cdot \left(\cos\left(\gamma_{1}\left(\frac{0\cdot 2\pi }{q}\right)\right)-\text{j}\cdot \sin\left(\gamma_{1}\left(\frac{0\cdot 2\pi }{q}\right)\right)\right)\\ +\left(\cos\left(n\left(\frac{1\cdot 2\pi }{q}\right)\right)+\text{j}\cdot \sin\left(n\left(\frac{1\cdot 2\pi }{q}\right)\right)\right)\\ \cdot \left(\cos\left(\gamma_{1}\left(\frac{1\cdot 2\pi }{q}\right)\right)-\text{j}\cdot \sin\left(\gamma_{1}\left(\frac{1\cdot 2\pi }{q}\right)\right)\right)+\cdots \\ +\left(\cos\left(n\left(\frac{\left(q-1\right)\cdot 2\pi }{q}\right)\right)+j\cdot \sin\left(n\left(\frac{\left(q-1\right)\cdot 2\pi }{q}\right)\right)\right)\\ \cdot \left(\cos\left(\gamma_{1}\left(\frac{\left(q-1\right)\cdot 2\pi }{q}\right)\right)-\text{j}\cdot \sin\left(\gamma_{1}\left(\frac{\left(q-1\right)\cdot 2\pi }{q}\right)\right)\right)] \end{array}$$

In this case, to excite the structural mode +n, the following equation has to be accomplished:

(14) $$n=\gamma_{1}\pm \lambda q$$

As for the case that the disk rotates in air, for one exciter the structural mode +n is excited with any excitation shape *γ*_1_. Nevertheless, when the disk rotates in water the structural mode +n is excited for an arbitrary number of exciters only if *γ*_1_ = *n*, which means that the excitation has to coincide with the structural mode shape in its shape and in its direction. When *γ*_1_ ≠ *n*, the structural mode +n is only excited if the number of exciters accomplish Equation (14).

## 3. Experimental Tests

### 3.1. Test Rig Setup

**Figure 5.**
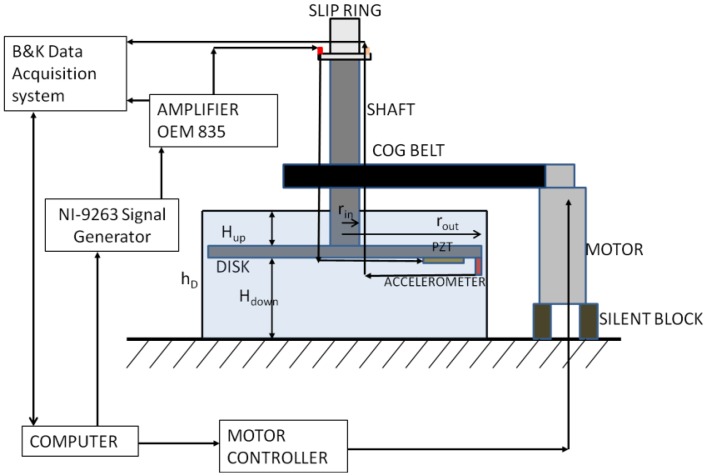
Rotating disk test rig.

To study the dynamic behavior of a rotating disk structure (rotating in air and water) due to the excitation characteristic of the rotor-stator interaction experimentally, a rotating test rig setup has been developed. It consists of a disk connected to a variable speed motor. The disk rotates up to 8 Hz in air and in water. When the disk is rotating, it is excited with several excitation patterns created with four piezoelectric patches PI-876A12 attached in two different configurations (only one PZT is shown in Figure 5). The patches work in a range of −100 V to 250 V. The excitation signals are created with a NI-9263 Signal Generator (National instruments, Austin, TX, USA) using a Labview^®^-code. Four independent signals can be generated with this generator in a range of −10 V to 10 V. To work in the PZTs range an amplifier OEM 835 with gain 25 is used. This amplifier sends the signals to the PZTs and also to the Bruel & Kjaer Acquisition System (Naerum, Denmark).

The response is measured with miniature and submergible Dytran 3006-A accelerometers (Dytran, Chatsworth, CA, USA), that are screwed on the disk. The added mass of the sensors and actuators is less than 1% of the mass of the disk, so it is checked that after installation of them, the vibration characteristics of the disk do not vary.

All the excitation and response signals of the rotating frame are transmitted to the stationary frame through a Michigan S10 slip ring system. This system is attached at the tip of the shaft. Ten independent circuit slip rings are used to transmit the signals. Since one channel of a sensor or exciter consist in a positive and a negative line, only four accelerometers and four patches can be used simultaneously. The negative terminals of the accelerometers are connected to one common point and the negative terminals of the patches are connected to another common point. The eight channels left are used to connect the four positve lines of the accelerometers and the four positive lines of the patches.

The rotating speed of the motor (Mavilor MLV-072, Santa Perpétua de Mogoda, Spain) is controlled with a computer. The vibrations of the motor are isolated from the rest of the test rig through a silent block. A scheme of the test rig used in this study is shown in Figure 5.

### 3.2. Rotating Disk and Piezoelectric Patches

**Figure 6.**
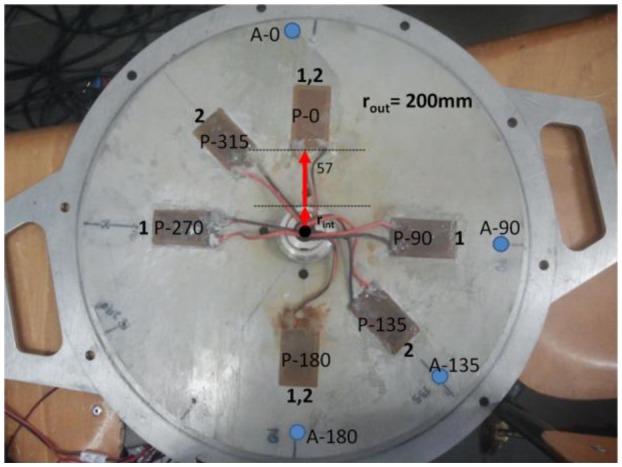
Rotating disk with installed PZTs and accelerometers.

The disk is made of stainless steel with an external radius r_out_ = 200 mm and a thickness of h_D_ = 8 mm. The disk has a hole on its center in order to attach the shaft. The nomenclature used for the patches is P-X, where X is the angle related to the 0° direction in counterclockwise direction, when the disk is attached to the shaft and viewing the test rig from the top. The same nomenclature is used for the accelerometers, with A-X. Four accelerometers (A-0, A-90, A-135, A-180) are screwed on the disk to measure the response. Six patches PI-876A12 (61 mm × 35 mm) are glued on the disk (P-0, P-90, P-135, P-180, P-270, P-315) to make the excitation, but only four can be used simultaneously cause the limited number of channels in the slip ring system. In order to excite properly the studied mode shapes they will be used in two different configurations shown in Figure 6. Configuration 1 is (P-0, P-90, P-180, P-270) and configuration 2 is (P-0, P-135, P-180, P-315). The mass of the disk is approximately 7.6 kg.

### 3.3. Proccedure

The main objective of the tests is to study the response of the disk in resonance, when it is excited with different rotating excitation patterns that simulate the RSI excitation. This is done exciting the first several natural frequencies of the rotating disk in air and water with different excitation patterns γ created with PZTs. The following procedure is applied:

(1) Firstly, only one patch is used with a sweep signal that excites the first several natural frequencies of the disk. This is done for the disk rotating in air and the disk rotating in water.
(2) It is checked that, when using the same excitation signal for two different patches the response of the contiguous accelerometer is different in terms of amplitude and phase, since the excitation depends on the mounting condition of the patch. Furthermore, for the same patch the relationship force/voltage changes within the excited frequency. Therefore patches have to be calibrated, in order to make a compensated excitation shape at one desired frequency. In this case, since the response of the disk is studied under resonance condition, the calibrated frequencies are the natural frequencies of the disk. For the first several natural frequencies, patches are calibrated to make a compensated excitation shape in angle and phase.
(3) Once patches are calibrated, the disk is excited with several rotating excitation patterns at the studied natural frequencies.

#### 3.3.1. Excitation with One Patch

Natural frequencies of the rotating disk in air and in water have to be determined. Since, the first several natural frequencies are the most relevant in the real case, cause they can be excited by RSI phenomena [14], this study is concerned in a frequency range of 0–1200 Hz. Therefore, for this disk a sweep signal from 0 to 1200 Hz is used to excite the first natural frequencies of the disk in air and in water. Such a signal can be described as:

(15) $$\begin{array}{cc} y=\mathrm{Asin}\left(\omega t\left(t\right)\right) & \text{for}0<t<t_{\text{end}} \end{array}$$

When a patch works with this signal, it excites all the frequency band from 0 Hz to $\frac{\omega t_{\text{end}}}{2\pi }$Hz. $\frac{\omega t_{\text{end}}}{2\pi }$ is selected as 1200 Hz in this case. *ω* (sweep rate) has to be enough small (slow sweep) in order to have a good resolution in frequency when applying the FFT, without losing information.

**Figure 7.**
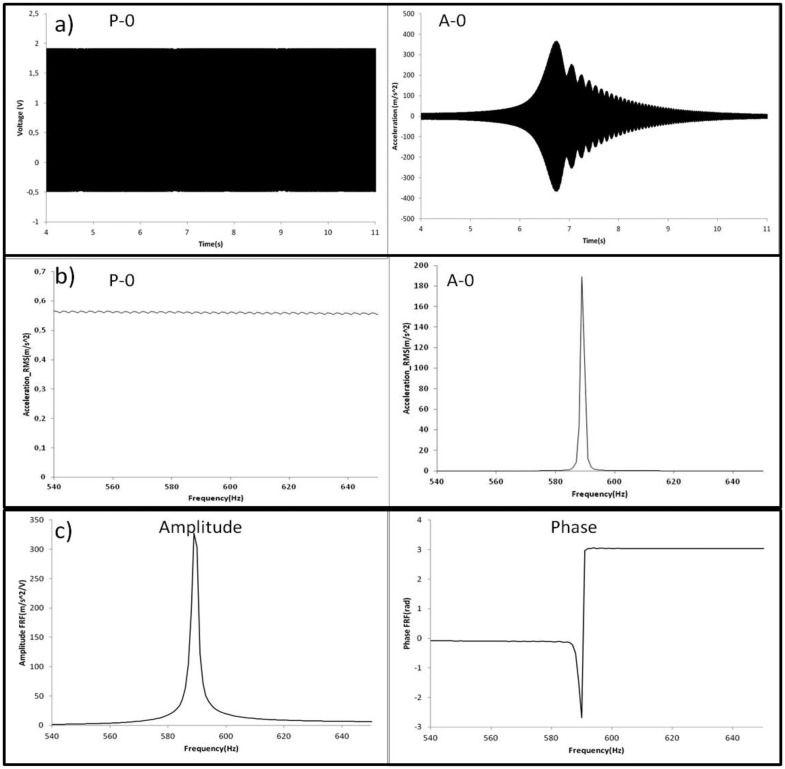
Determination of the natural frequencies of the rotating disk. (**a**) Time signals; (**b**) Signals after FFT; (**c**) FRF Amplitude and Phase.

In Figure 7, the procedure to obtain the natural frequencies of the disk is shown for one resonance and one sensor. The time signal of the excitation P-0 (Figure 7a) shows a slow sweep excitation. The time signal of A-0 (Figure 7a) shows that a resonance occur at certain time.

To obtain the frequency content of these signals, a Hanning Window of 4 s (resolution 0.25 Hz) is applied on the time signals. Since this window is shorter than the total length of the time signals, it is translated 0.2 s (5% of the window length) every average. In each average, the FFT is applied in both signals and superposed to the other averages with the maximum hold method, which considers only the maximum value for each frequency. In this way the frequency content of both signals is obtained (Figure 7b). Using both response (A-0) and excitation (P-0) signals, the frequency response is obtained (Figure 7c). Natural frequencies are detected in precision looking at the peaks of the frequency response function (FRF). The corresponding mode shapes are obtained analyzing the relative phase of the four accelerometers (Figure 6) and contrasting with the analytical model. In this way, natural frequencies and mode shapes are determined for the still and rotating disk in air and in water.

#### 3.3.2. Calibration of the Patches

A closer view from the disk with the installed piezoelectric patches can be seen in Figure 6. The nomenclature used for the patches is P-X, where X is the angle related to the 0° direction in counterclockwise direction, when the disk is attached to the shaft and viewing the test rig from the top. The same nomenclature is used for the accelerometers, with A-X.

The relation force/voltage characteristic for the piezoelectric patches changes for each patch (since it depends on the mounting condition of the patch) and with the signal frequency. To make that patches work with the same amplitude (in force) and with the desired phase to each other, they have been previously calibrated at the natural frequencies studied. Here is explained how the calibration is performed for the mode n = ±2 (disk rotating in air) and configuration 1 (Figure 6). For other modes and configurations the procedure is equivalent.

First, only patch P-0 is used at one natural frequency with a peak value of 75 V. (X_A-0(P-0)_)_fn_ (amplitude of the vibration of accelerometer A-0 due to an excitation with P-0 at the natural frequency n) and (α_A-0(P-0)/P-0_)_fn_ (angle between the signal of A-0 and the signal of P-0 due to an excitation with P-0 at the natural frequency n) are measured.

When using another patch at the same natural frequency, (X_A-i(P-i)_)_fn_ and (α_A-i(P-i)/P-0_) _fn_ are measured (i is 90°, 180° and 270° in this case). The amplitude of P-i is changed in order to accomplish (X_A-i(P-i)_)_fn_=(X_A-0(P-0)_)_fn_ for each i. Also a phase shift between signal P-i and signal P-0 is introduced to make that (α_A-i(P-i)/P-0_)_fn_=(α_A-0(P-0)/P-0_)_fn_. In this case the signal of P-0 is acquired as a reference, but is not really used to excite the patch P-0. In the specified case, the calibration of the patches has been done adjusting the signals of the patches, to accomplish:

(16) $$\begin{array}{c} {\left({\text{X}}_{\text{A}-0\left(\text{P}-0\right)}\right)}_{\text{fn}}={\left({\text{X}}_{\text{A}-90\left(\text{P}-90\right)}\right)}_{\text{fn}}={\left({\text{X}}_{\text{A}-180\left(\text{P}-180\right)}\right)}_{\text{fn}}={\left({\text{X}}_{\text{A}-90\left(\text{P}-270\right)}\right)}_{\text{fn}}\\ {\left(\alpha_{\text{A}-0\left(\text{P}-0\right)/\text{P}-0}\right)}_{\text{fn}}={\left(\alpha_{\text{A}-90\left(\text{P}-90\right)/\text{P}-0}\right)}_{\text{fn}}={\left(\alpha_{\text{A}-180\left(\text{P}-180\right)/\text{P}-0}\right)}_{\text{fn}}={\left(\alpha_{\text{A}-90\left(\text{P}-270\right)/\text{P}-0}\right)}_{\text{fn}} \end{array}$$

**Figure 8.**
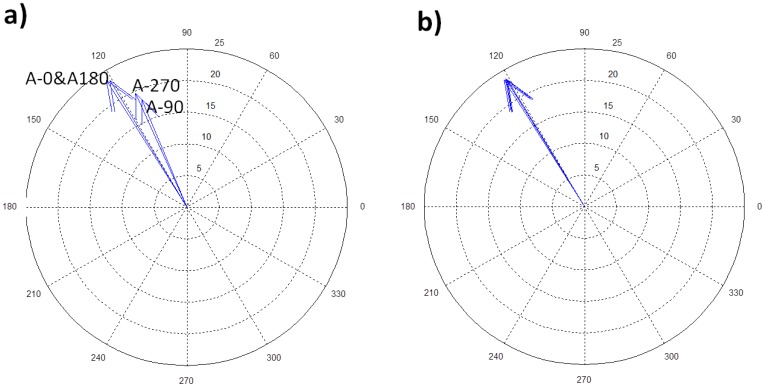
Polar plot of the sensors. (**a**) Before calibration of PZTs; (**b**) After calibration of PZTS.

The accomplishment of Equation (16) for each f_n_ (f_n_ are the first natural frequencies of the disk), guarantee that patches are properly calibrated in amplitude and phase (Figure 8b). After patches are calibrated, a phase shift between them (apart from the phase shift introduced for the calibration) can be introduced to make the desired excitation pattern.

#### 3.3.3. Rotating Excitation at the First Natural Frequencies

**Figure 9.**
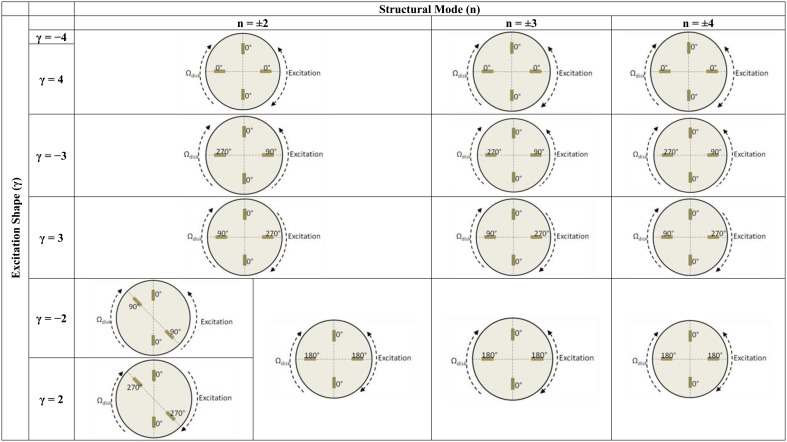
Excitation patterns created with the installed Piezoelectric Patches.

After the first several natural frequencies are obtained and patches are calibrated to make a compensated excitation shape in these frequencies, the disk is excited with several rotating excitation patterns that simulate the RSI that occurs in real turbomachinery. This is performed for the disk rotating in air and for the disk rotating in water. The excitation patterns that are created with four patches installed in the two different configurations shown in Figure 6 are represented in Figure 9. The response is measured with the accelerometers screwed on the disk.

As Figure 9 shows, with four patches attached at 90° it is not possible to define the rotating direction of the excitation for the modes n = ±2 and n = ±4, changing the phases between exciters. For n = ±3 this direction is decided changing the phase of the patches (Figure 9). For n = ±2 another configuration is tested (P-0, P-135, P-180 and P-315), that defines the rotating direction. With this configuration is also not possible to define the direction of the excitation for n = ±4.

## 4. Results and Discussion

### 4.1. Natural Frequencies of the Disk

The first several natural frequencies of the disk are determined for the case that the disk is rotating in air and in water. To determine these natural frequencies and mode shapes, two methods are used, the analytical method presented in Section 2 and the experimental method explained in Section 3. For the rotating case in air and water, the maximal velocity will be studied in this paper, *i.e.*, when the disk is rotating at 8 Hz.

#### 4.1.1. Analytical

To determine the natural frequencies in air for each structural mode ±n, the density, thickness and averaged radius are necessary (Equation (5)). The parameter D* is determined using the methodology presented in [25]. In that study only the formulas for n < 3 are presented. Therefore, the selected mode to determine D* is n = ±2. Once D* is obtained, the modes n = ±3 and n = ±4 are determined for the non rotating disk. These are the predicted natural frequencies below 1.2 kHz, which is the range that will be studied in this paper. According to the analytical model for low rotating speeds of the disk the rotation does not affect in Equation (5). Therefore the natural frequencies calculated for the non rotating case are also the natural frequencies for the disk rotating at maximal speed of 8 Hz.

When the disk is submerged in water, apart from the parameters used in the case of air, the density of water, the distances to the rigid walls H_up_ & H_down_ and the velocity of the fluid with respect to the disk are needed (Equation (6)). To estimate the averaged values of Ω_up_ and Ω_down_, a CFD calculation has been performed. This calculation gives the velocity of the particles of fluid with respect the stationary frame. An averaged value for the rotating speed of the fluid particles with respect to the disk for the upper and lower field (Figure 1) is calculated. For the rotating speed of the disk of 8 Hz it is determined that Ω_up_ = 4.97 Hz and Ω_down_ = 4.92 Hz. All the parameters used in Equation (6) (and also in Equation (5)) are presented in Table 1.

#### 4.1.2. Experimental

**Figure 10.**
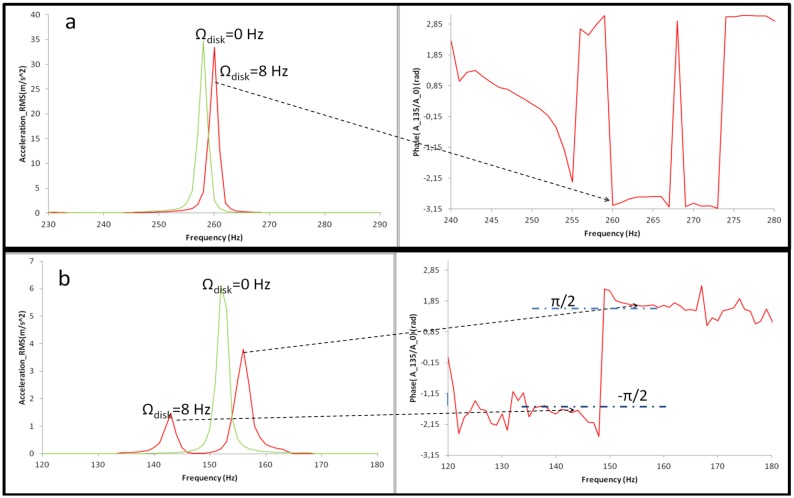
(**a**) Natural frequencies n = ±2 of the rotating disk in air at Ω_disk_ = 0 Hz and Ω_disk_ = 8 Hz; (**b**) Natural frequencies n = ±2 of the disk rotating in water at Ω_disk_ = 0 Hz and Ω_disk_ = 8 Hz.

From the experimental point of view, the disk is excited with only one patch for the stationary case and for the rotating case in air and in water. The way to obtain the natural frequencies of the disk below 1.2 kHz in all these cases is mentioned in Section 3. Figure 10a shows the natural frequency n = ±2 of the disk in air for the stationary and for the rotating case and Figure 10b shows the same for the case that the disk is rotating in water.

It is seen that for the case in air only a slight increase in the natural frequency is produced when the disk is rotating in air. This increase represents less than 1% for the studied modes. The phase shift between sensors (Figure 10a) shows that the corresponding mode shape is a standing wave (viewed from the rotating frame) with all the points moving in phase (0 rad) or in counterphase (π rad) to each other. For the case that the disk rotates in water, an important difference is observed. In this case two peaks are detected (from the rotating frame), which correspond to the modes n = 2 and n = −2. These are two travelling waves travelling in the opposite direction as the analytical model predicts, and the phase of accelerometers confirms (Figure 10b). According to Equation (3), the phase shift between two points with a spatial phase shift of Θ, have a phase difference of ±nΘ, depending on the mode shape and on the travelling direction. Therefore, for the shown accelerometers in Figure 10b (A-0 & A-135), this phase shift has to be ±π/2. The values of the natural frequencies of the disk below 1.2 kHz in all the cases for analytical and experimental methods are shown in Table 2.

Differences of less than 5% are obtained when comparing both methods**.** After the first several natural frequencies of the disk are obtained, patches are calibrated to make the desired excitation patterns in these natural frequencies.

### 4.2. Dynamic Behavior of the Rotating Disk in Air Due to an RSI

The RSI is the superposition of excitations that change in frequency when increasing the rotating speed of the rotor (term *lZ_o_*Ω_rot_ of Equation (8)) and with an excitation moving with respect the rotating disk (term *kZ_b_* − *lZ_o_* of Equation (8)). In this paper, the dynamic behavior of the disk when the excited frequency coincides with a natural frequency of the disk is studied. A slow sweep signal around the resonance with the patterns specified in Figure 9 is applied on the disk. Amplitudes of vibration of the accelerometer A-0 (attached on the rotating disk) under resonance condition are studied for all the cases represented in Figure 9.

#### 4.2.1. Analytical

Applying Equation (11) particularizing the excitation shape and the structural mode shape the amplitude of the disk under different resonance situations is determined.

#### 4.2.2. Experimental

**Figure 11.**
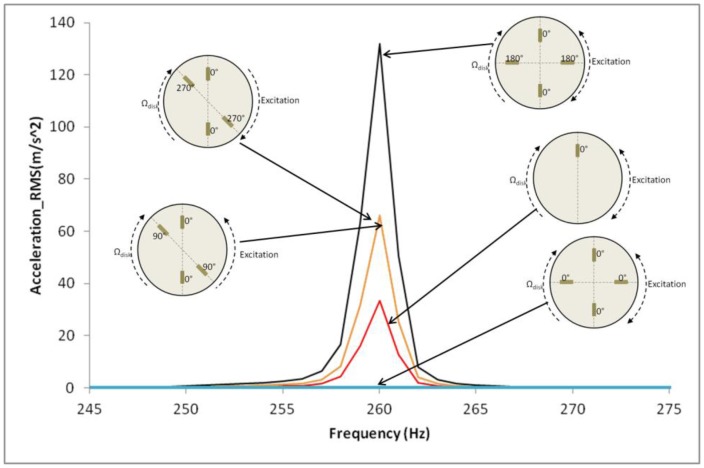
Resonances around n = ±2 (experimental) for the disk rotating in air (Ω_disk_ = 8 Hz). Different excitation patterns.

Once natural frequencies are determined and patches are calibrated, the disk is excited with a sweep signal passing through the resonance with the excitation patterns presented in Figure 9. As Figure 11 shows, for n = ±2 when the disk is excited with the pattern γ = ±2 the resonance is amplified and when it is excited with γ = ±4 is eliminated (as predicted in Equation (12)). Note that for the symmetric-position of patches the excitation direction for γ = ±2 and γ = ±4 cannot be defined. For the same structural mode, the disk is excited with the non-symmetric position of the PZT. In this case the direction of excitation can be defined for γ = ±2. As Figure 11 shows, both excitations amplify the amplitude of resonance, as the mode shape is a standing wave on the disk.

The rest of resonance amplitudes divided by the amplitude of resonance due to one patch excitation (red line in Figure 11) are presented in Table 3 compared with the analytical results. A difference of less than 1.5% between the analytical model and experimental results is obtained for all the experimented cases. From the experimental results can be extracted that patches are feasible to excite a thick disk in air and that RSI excitation can be studied and simulated (or attenuated) with PZTs. It is found that for each mode n, only one resonance (from the rotating system) is produced and amplified when the excitation shape γ coincides with the structural mode shape n, with no matter of the direction of rotation of excitation. For the other excitation shapes γ, with the positions of the exciters used, the amplitude of resonance is almost 0.

### 4.3. Dynamic Behavior in Water Due to an RSI

#### 4.3.1. Analytical

Applying Equation (14) particularizing the excitation shape and the structural mode shape the amplitude of the disk under different resonance situations is determined.

#### 4.3.2. Experimental

**Figure 12.**
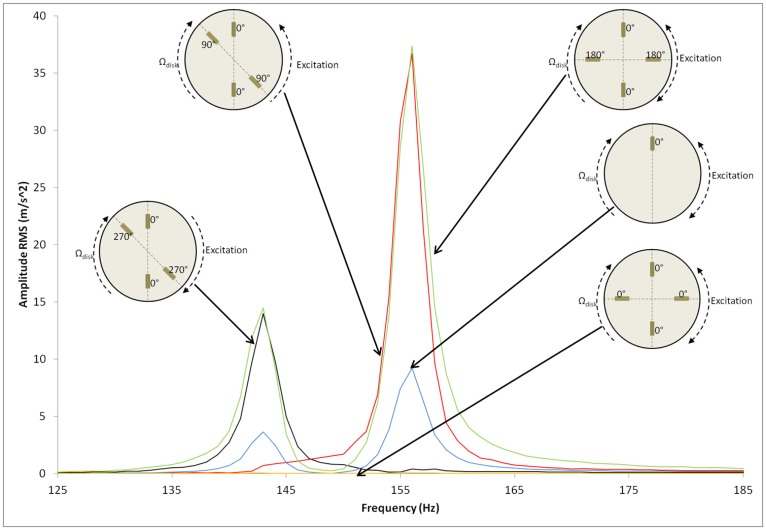
Resonances around n = 2 and n = −2 (experimental) for the disk rotating in water (Ω_disk_ = 8 Hz). Different excitation patterns.

Again the same experimental procedure explained in Section 3 is applied when the disk is rotating in water. Figure 12 shows the excitation of the structural modes n = +2 and n = −2 with different excitation patterns.

In this case, when the disk is excited with one patch, the two resonances (n = 2 and n = −2) are detected from the rotating frame. For the excitation γ = ±2 without defining the rotating direction of the excitation, both resonances are amplified. When γ is different than ±2, the amplitude of the resonance is almost 0 as predicted in Equation (14) for the position of exciters used. Only one resonance appears if the rotating direction of the excitation is defined. In the case of the non-symmetric position of patches this direction is defined (Figure 9) and it can be checked that to excite the structural mode n = 2 the excitation γ = 2 is necessary. For the same structural mode the excitation γ = −2 eliminates the resonance. The same conclusion is achieved for the structural mode n = −2, which needs an excitation shape γ = −2 to be amplified. The rest of resonance amplitudes compared to the case of one patch excitation are presented in Table 4 compared with the results of the analytical model.

A difference of less than 4% between methods is obtained for all the tested cases. From experimental results can be extracted that patches are feasible to excite a thick disk in water and that the RSI can be studied and simulated (or attenuated) with PZT. To notice is that the disk has two natural frequencies for each mode ±n (viewed from the rotating system) when it rotates in water. These are detected when excited with one patch. The resonance is amplified only in case that the excitation shape γ coincides with the structural mode n in magnitude and rotating direction. Both resonances are amplified when the rotating direction of the excitation shape is not defined. For other excitation patterns (n ≠ γ), with the used exciters, both resonances are eliminated.

## 5. Conclusions

The dynamic behavior of a thick disk rotating in air and inside a casing filled with water has been analyzed experimentally and analytically. The disk has been excited with several rotating excitation patterns simulating the rotor-stator interaction (RSI) excitation.

For an accurate analysis of the disk behavior both exciters and sensors measuring the response were located on the disk (rotating frame). For the excitation several PZT actuators attached to the disk were used. PZTs do not affect the mass of the disk and do not perturb the flow of water produced by the rotation of the disk inside the casing. PZT actuators have been used several times to excite thin rotating disks in air and from the stationary frame, but never to excite a thick disk submerged in water and confined inside a casing. Experiments presented have demonstrated that it is feasible to use PZT actuators in thick structures in air and submerged in water. This is interesting because PZTs could be used to determine the dynamic response of disk-like structures, such as turbomachinery impellers in actual operating conditions.

Natural frequencies of the rotating disk in air and in water have been determined. The effect of rotation is very important when the disk rotates in water. Viewed from the rotating frame, even for low rotation speeds (8 Hz), for each natural frequency found in the non rotating case (one for each diametrical mode), two natural frequencies that correspond to two travelling waves rotating in the opposite direction appear. For the same rotating speed in air, only one natural frequency that correspond to a standing wave (viewed from the rotating frame) is observed. This behavior of the natural frequencies and mode shapes (when the disk rotates in air and in water) is determined experimentally and predicted by the analytical model used.

The dynamic response of the rotating disk in water at one natural frequency depends on the excitation shape and also on the rotating direction of the excitation. When the excitation is fixed on the rotating frame, the response at the two natural frequencies of the corresponding diametrical mode is amplified. However when the excitation spins in the same direction of the disk only the lower natural frequency is excited, which corresponds to the travelling wave travelling in the same direction as the disk. When the excitation spins in the opposite direction only the higher natural frequency is excited, which corresponds to the travelling wave travelling in the opposite direction. If the excitation shape does not coincide with the diametrical mode considered, the response at both natural frequencies is almost zero (for the studied configuration of patches). When the disk rotates in air, only the excitation shape affects the dynamic response. If the excitation shape coincides with a diametrical mode, the response of the disk is amplified at the corresponding natural frequency, with no matter of the rotating direction of the excitation (rotating with the disk, counterwise or standing). These results are obtained experimentally and using the analytical model.

## Figures and Tables

**Table 1.** Parameters used to determine the natural frequencies of the rotating disk in air and in water (Ω_disk_ = 8 Hz) for each n.

**Longitudinal Parameters (m)**				**Densities (Kg/m^3^)**		**Rotating Velocities (Hz)**		**Bending Stiffness (N/m)**
r_o_	H_up_	H_down_	h_D_	ρ_D_	ρ_water_	Ω_up_	Ω_down_	D*
0.072	0.03	0.077	0.008	7800	1000	4.97	4.92	253

**Table 2.** Natural frequencies (Hz) of the stationary and rotating disk in air and water. Analytical and experimental values.

		**Air**			**Water**		
		**Analytic**	**Experim.**	**Error**	**Analytic**	**Experim.**	**Error**
**Ω_disk_ = 0 Hz**	**n = ±2**	260.03	257.75	0.9%	151.37	150.50	0.6%
	**n = ±3**	585.07	588.25	0.5%	393.20	383.25	2.5%
	**n = ±4**	1040.13	1031.50	0.8%	750.85	733.25	2.3%

**Ω_disk_ = 8 Hz**	**n = ±2**	260.03	259.75	0.1%	145.33	143.25	1.4%
					157.14	156.00	0.7%
	**n = ±3**	585.07	590.25	0.9%	386.23	374.00	3.1%
					399.62	390.00	2.4%
	**n = ±4**	1040.13	1033.25	0.7%	743.25	708.75	4.6%
					757.89	729.50	3.7%

**Table 3.** Amplification of the resonances (A_γ_/A_1-PATCH_) of the rotating disk in air (Ω_disk_ = 8 Hz) due to the different excitation patterns. Analytical, experimental and error.

	**Excitation Shape (γ)**							

		**γ = 2**		**γ = −2**	**γ = 3**	**γ = −3**	**γ = 4**	**γ = −4**
**Structural Mode (n)**	**n = ±2**	2		2				
		1.981		1.971				
		1.0%		1.5%	0	0		0
			4		0.003	0.004	0.003	
			3.942		-	-	-	
			1.5%					

	**n = ±3**		0		2	2	0	
			0.003		2.005	2.008	0.005	
			-		0.3%	0.4%	-	

	**n = ±4**		0		0	0	4	
			0.017		0.003	0.001	4.042	
			-		-	-	1.1%	

**Table 4.** Amplification of the resonances (A_γ_/A_1-PATCH_) of the rotating disk in water (Ω_disk_ = 8 Hz) due to the different excitation patterns. Analytical, experimental and error.

	Excitation Shape (γ)							

		γ = 2		γ = −2	γ = 3	γ = −3	γ = 4	γ = −4
Structural Mode (n)	**n = +2**	4		0				
		3.852		0.120				
		3.7%		-	0	0		0
			4		0.007	0.008		0.003
			3.983		-	-		-
			0.4%					

	**n = −2**	0		4				
		0.033		3.991				
		-		0.2	0	0		0
			4		0.003	0.004		0.015
			4.101		-	-		-
			3%					

	**n = +3**		0		4	0		0
			0.003		4.023	0.092		0.007
			-		0.6%	-		-
	**n = −3**		0		0	4		0
			0.002		0.025	3.965		0.005
			-		-	0.9%		-

	**n = ±4**		0		0	0		4
			0.005		0.009	0.007		4.152
			-		-	-		3.8%

	**n = −4**		0		0	0		4
			0.003		0.008	0.017		4.102
			-		-	-		2.6%

## References

[1] Jacquet-Richardet G., Torkhani M., Cartraud P., Thouverez F., Nouri T., Herran M., Gibert C., Baguet S., Almeida P., Peletan L. (2013). Rotor to stator contacts in turbomachines. Review and application. Mech. Syst. Signal Process..

[2] Shlyannikov V.N., Iltchenko B.V., Stepanov N.V. (2001). Fracture analysis of turbine disks and computational-experimental background of the operational decisions. Eng. Failure Anal..

[3] Egusquiza E., Valero C., Huang X., Jou E., Guardo A., Rodriguez C. (2012). Failure investigation of a large pump-turbine runner. Eng. Failure Anal..

[4] Campbell W. (1924). The protection of steam turbine disk wheels from axial vibration. Trans. ASME.

[5] Lamb H., Southwell R.V. (1921). The vibrations of spinning discs. Proc. R. Soc. Lond..

[6] Southwell R.V. (1922). On the free transverse vibrations of a uniform circular disc clamped at its centre and on the effects of rotation. Proc. R. Soc. Lond..

[7] Tobias S.A., Arnold R.N. (1957). The influence of dynamical imperfection on the vibration of rotating disks. Proc. Inst. Mech. Eng..

[8] Mehdigholi H. (1991). Forced Vibration of Rotating Discs and Interaction with Non-Rotating Structures. Ph.D. Thesis.

[9] Ahn T.K., Mote C.D. (1998). Mode identification of a rotating disk. Exp. Mech..

[10] Heo J.W., Chung J. (2004). Vibration analysis of a flexible rotating disk with angular misalignment. J. Sound Vib..

[11] Bauer H.F., Eidel W. (2007). Transverse vibration and stability of spinning circular plates of constant thickness and different boundary conditions. J. Sound Vib..

[12] Pust L., Pesek L. (2011). Vibration of circular bladed disk with imperfections. J. Bifurc. Chaos.

[13] Pust L., Pesek L. (2011). Vibration of imperfect rotating disk. Appl. Comput. Mech..

[14] Kubota Y., Ohashi H. A study on the natural frequencies of hydraulic pumps.

[15] Parker R., Watson J. (1972). Interaction effects between blade rows in turbomachines. Proc. Inst. Mech. Eng..

[16] Dring R., Joslyn H., Hardin L., Wagner J. (1982). Turbine rotor-stator interaction. ASME J. Eng. Power.

[17] Nicolet C., Ruchonnet N., Avellan F. One-dimensional modeling of rotor stator interaction in francis turbine.

[18] Zobeiri A., Kueny J.-L., Farhat M., Avellan F. Pump-turbine rotor-stator interactions in generating mode: Pressure fluctuation in distributor channel.

[19] Yang Z., Guo S., Yang J., Hu Y. (2009). Electrically forced vibration of an elastic plate with a finite piezoelectric actuator. J. Sound Vib..

[20] Gomis-Bellmunt O., Ikhouanne F., Montesinos-Miracle D. (2009). Control of a piezoelectric actuator considering hysteresis. J. Sound Vib..

[21] Cheng C.C., Lin C.C. (2005). An impedance approach for vibration response synthesis using multiple PZT actuators. Sens. Actuators A Phys..

[22] Sekouri E.M., Hu Y.-R., Ngo A.D. (2004). Modeling of a circular plate with piezoelectric actuators. Mechatronics.

[23] Wang X., Huang Z. (2006). Feedback control and optimization for rotating disk flutter suppression with actuators patches. AIAA J..

[24] Yan T., Xu X., Han J., Lin R., Ju B., Li Q. (2011). Optimization of sensing and feedback control for vibration/flutter of rotating disk by PZT actuators via air coupled pressure. Sensors.

[25] Belvins R. (1984). Formulas for Natural Frequency and Mode Shape.

[26] White F. (1979). Fluid Mechanics.

[27] Singh K., Mallik A.K. (1977). Wave propagation and vibration response of a periodically supported pipe conveying fluid. J. Sound Vib..

[28] Wood G.M., Knudsen L.K., Hammitt F.G. (1967). Cavitation damage studies with rotating disk in water. J. Fluids Eng..

[29] Zhiye J. Experimental investigation on cavitation and cavitation erosion with rotating disk.

[30] De La Torre O., Escaler X., Egusquiza E., Farhat M. (2013). Experimental investigation of added mass effects on a hydrofoil under cavitation conditions. J. Fluids Struc..

[31] Askari E., Jeong K.-H., Amabili M. (2013). Hydroelastic vibration of circular plates immersed in a liquid-filled container with free surface. J. Sound Vib..

[32] Heylen W. (2007). Modal Analysis Theory and Testing.

